# Environmental Pollution and Its Impact on Kidney Diseases: A Comprehensive Review of Current Evidence

**DOI:** 10.3390/life16020291

**Published:** 2026-02-08

**Authors:** Seung Eun Lee, Yong Seek Park

**Affiliations:** Department of Microbiology, College of Medicine, Kyung Hee University, Seoul 02447, Republic of Korea; eunlee@khu.ac.kr

**Keywords:** kidney disease, environmental pollution, oxidative stress, inflammation

## Abstract

Kidney disease is a growing global public health challenge that accounts for substantial morbidity, premature mortality, and rising healthcare costs. Although diabetes mellitus and hypertension remain the principal clinical risk factors for renal injury, accumulating evidence indicates that environmental pollution represents an independent and globally pervasive contributor to kidney disease burden. Long-term exposure to environmental toxicants, including heavy metals, ambient air pollutants, persistent organic pollutants, and endocrine-disrupting chemicals, has been consistently associated with acute kidney injury, an accelerated decline in renal function, and progression to end-stage kidney disease. The kidney is characterized by a high perfusion rate, specialized tubular transport systems, and a central role in xenobiotic metabolism and excretion, which confer heightened vulnerability to environmental insults. Experimental and epidemiological studies have demonstrated that pollutant exposure across the life course converges on shared pathogenic mechanisms, including oxidative stress, inflammatory signaling, mitochondrial dysfunction, fibrogenesis, and persistent epigenetic alterations. Importantly, environmental toxicants not only initiate renal injury, but they also impair intrinsic repair processes, exacerbating susceptibility to chronic and progressive kidney disease. This Review integrates population-based epidemiological data with experimental mechanistic evidence to define environmental exposures, renal cellular targets, and convergent molecular pathways underlying pollutant-induced nephrotoxicity, and aims to translate this knowledge into actionable strategies for kidney disease prevention, clinical risk stratification, and evidence-informed environmental policy.

## 1. Introduction

Kidney diseases encompass a spectrum of clinical entities ranging from acute kidney injury to chronic kidney disease (CKD) and end-stage kidney failure. Together, these conditions represent some of the fastest-growing contributors to global morbidity and mortality worldwide [1,2]. Current estimates indicate that CKD affects approximately 800 million adults, with its prevalence more than doubling since 1990 [1]. Kidney dysfunction contributes not only to deaths attributable to renal failure, but also to a substantial fraction of cardiovascular mortality. This positions kidney disease as a major determinant of systemic health rather than an isolated organ pathology [2].

A growing body of evidence has demonstrated that even modest or transient declines in kidney function have lasting consequences. Individuals who experience mild reductions in glomerular filtration rate face persistently elevated risks of CKD progression, major cardiovascular events, and premature death [3,4,5,6]. These observations have reframed kidney disease as a powerful amplifier of systemic vulnerability capable of reshaping long-term health trajectories, even when the initial injury appears clinically limited.

Despite the notable advances in the prevention and treatment of diabetes, hypertension, and other established risk factors, the global burden of kidney disease continues to rise [1,7]. There is pronounced geographic heterogeneity in incidence and progression, as well as enduring socioeconomic and racial disparities. These patterns are not fully explained by traditional determinants, such as metabolic diseases or inherited susceptibility alone [8,9]. These patterns suggest the presence of additional population-level drivers that operate across diverse settings and life stages, thus highlighting critical gaps in current risk models.

Environmental pollution has emerged as a plausible and increasingly compelling contributor to this unexplained risk. In contrast to classical risk factors, environmental exposures are pervasive, largely involuntary, and cumulative, affecting individuals across the entire life course and disproportionately burdening vulnerable populations [10,11,12]. The kidneys are particularly susceptible to environmental toxicants owing to their high perfusion rate, concentrating capacity, and central role in xenobiotic filtration and excretion. Chronic exposure to low levels of toxicants can result in sustained renal stress, especially in the context of aging, metabolic diseases, or reduced nephron reserves [13,14].

Environmental toxicants rarely act in isolation as nephrotoxic agents. Instead, they interact with established risk factors such as diabetes, hypertension, and vascular dysfunction. Through these interactions, environmental exposure lowers the threshold for kidney injury and accelerates maladaptive repair processes [15]. In this framework, pollution does not function as an isolated cause of kidney disease but rather as a risk multiplier that magnifies pre-existing biological susceptibility and contributes to marked interindividual heterogeneity in the disease course [16,17].

The temporal dimension of environmental exposure further complicates the risk for kidney disease. Exposure often begins during critical windows of development, including fetal life and early childhood. Experimental and epidemiological studies link early-life exposure to toxicants with reduced nephron endowment, which predisposes individuals to hypertension and CKD decades later [18,19,20,21]. At the opposite end of the life course, aging kidneys exhibit a diminished regenerative capacity and an increased susceptibility to cumulative injury, rendering them particularly vulnerable to repeated or chronic toxic insults [22].

**Figure 1:**
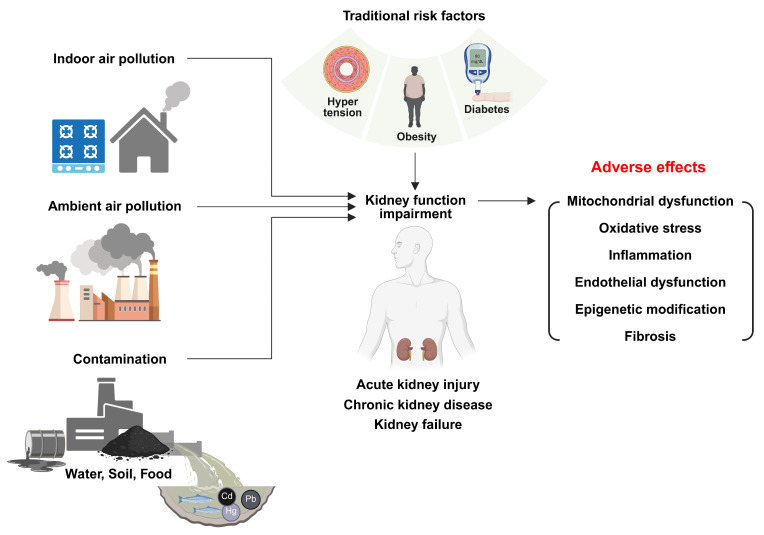
Integrative Framework of Environmental Nephrotoxicity: Convergent Pathways to Chronic Kidney Disease. Created in BioRender. Lee, SE. (2026) https://BioRender.com/k3itynv.

When taken together, these observations underscore the need to identify harmful environmental exposures and to delineate the cellular and molecular processes through which they shape renal injury, repair, and long-term functional decline. In this review, we compile and critically evaluate epidemiological and experimental evidence linking environmental pollution to kidney dysfunction and disease progression, with a focus on how sustained toxicant exposure intersects with aging, metabolic stress, and impaired regenerative capacity to drive kidney disease across the life course (Figure 1).

Beyond summarizing existing evidence, this review advances a unifying life course framework in which environmental pollution is positioned as an exposome-driven risk multiplier for kidney disease. By integrating chemically and physically diverse exposures within shared pathogenic pathways, such as oxidative stress, endothelial dysfunction, maladaptive repair, and fibrosis, we provide a conceptual explanation for population-level heterogeneity in kidney disease onset and progression.

## 2. Air Pollution and Kidney Diseases

Air pollution is increasingly recognized as a major environmental risk factor for kidney diseases, with effects observed across diverse populations, geographic regions, and stages of renal dysfunction [23,24]. Ambient air pollution, particularly fine particulate matter (PM_2.5_), is pervasive, impacting nearly the entire global population and contributing substantially to the overall burden of kidney diseases [25,26,27]. Recent epidemiological and experimental evidence has established air pollution as an independent risk factor for AKI, CKD, CKD progression, and kidney-related mortality [28,29,30].

### 2.1. Fine Particulate Matter (PM_2.5_ and PM_10_)

PM_2.5_ has emerged as the predominant air pollutant that is consistently associated with adverse kidney outcomes. Evidence from large-scale cohort studies and meta-analyses demonstrates that long-term PM_2.5_ exposure correlated with a reduced estimated glomerular filtration rate (eGFR), increased albuminuria, higher CKD incidence, and accelerated progression to kidney failure [27,30,31,32,33]. Likewise, short-term increases in PM_2.5_ concentrations have been linked to elevated risks of AKI-related hospitalizations and kidney-related mortality, highlighting both acute and chronic renal vulnerability to particulate exposure [28,29,34].

Importantly, several studies in the literature have reported monotonic exposure–response relationships without clear thresholds, suggesting that kidney injury may occur even at PM_2.5_, below the current air quality standards in many countries [35,36]. These findings raise critical concerns regarding the adequacy of the existing regulatory limits for kidney protection and underscore the relevance of air pollution as a public health target for CKD prevention.

### 2.2. Gaseous Air Pollutants

In addition to particulate matter, gaseous air pollutants, including nitrogen dioxide (NO_2_), sulfur dioxide (SO_2_), ozone (O_3_), and carbon monoxide (CO), have been associated with impaired kidney function. NO exposure, which is often used as a marker of traffic-related air pollution, has been linked to reduced eGFR and increased CKD prevalence in multiple epidemiological studies [37,38]. Ozone exposure has been associated with a higher CKD prevalence and kidney-related mortality, particularly in warmer climates where ozone formation is accelerated [39,40].

Although the renal effects of gaseous pollutants have been shown to be generally less pronounced than those of PM_2.5_, their widespread exposure and potential synergistic interactions with particulate matter may amplify overall nephrotoxicity at the population level [41,42].

### 2.3. Biological Mechanisms Linking Air Pollution to Kidney Injury

Past mechanistic studies have suggested that air pollution contributes to kidney disease through interconnected systemic and renal-specific pathways. Inhaled particles and gases induce pulmonary oxidative stress and inflammation. This leads to the systemic dissemination of proinflammatory cytokines, reactive oxygen species, and ultrafine particles [43,44]. These circulating mediators promote endothelial dysfunction, microvascular injury, and impaired renal autoregulation [45,46]. Together, these processes contribute to glomerulosclerosis and tubulointerstitial fibrosis.

Experimental models have further demonstrated that PM_2.5_ exposure activates renal inflammatory signaling pathways, increases oxidative stress within tubular epithelial cells, and disrupts mitochondrial function, resulting in tubular injury and fibrotic remodeling [47,48,49,50,51,52,53]. Epigenetic alterations and metabolic dysregulation induced by air pollution may contribute to long-term renal susceptibility [54].

### 2.4. Susceptible Populations and Effect Modification

However, the renal effects of air pollution are not uniformly distributed across populations. Individuals with pre-existing risk factors, including diabetes, hypertension, older age, and baseline CKD, exhibit heightened susceptibility to pollution-related renal decline [55,56]. Notably, emerging evidence has suggested that genetic predisposition modifies air pollution–associated kidney risk. Carriers of high-risk *APOL1* variants have demonstrated amplified associations between PM_2.5_ exposure, and CKD progression, highlighting gene–environment interactions in pollution-related nephropathy [57].

Socioeconomic disadvantages and residential proximity to pollution sources further exacerbate exposure and risk, reinforcing health inequities in kidney disease burden [58,59].

### 2.5. Public Health and Policy Implications

From a public health perspective, air pollution is a paradigmatic example of a policy-sensitive risk factor for kidney disease. Unlike many individual-level risk factors, exposure to ambient air pollution is largely involuntary and shaped by regulatory standards, energy policies, transportation systems, and urban design [35,41]. A recent meta-analysis suggested that long-term exposure to air pollutants can significantly accelerate renal dysfunction in individuals living in highly polluted areas, thereby amplifying the regional burden of kidney disease [33]. Despite this compelling body of evidence, kidney outcomes are rarely incorporated into air quality health impact assessments or regulatory decision making. Integrating kidney-specific endpoints into environmental policy frameworks could strengthen the rationale for stricter air quality standards and also catalyze cross-sectoral strategies for kidney disease prevention.

## 3. Heavy Metals and Metal Mixtures

Heavy metals are among the most extensively studied environmental nephrotoxins and are major contributors to the kidney disease burden worldwide. Human exposure to heavy metals occurs in both occupational and environmental settings because these elements are extensively utilized in industrial, domestic, agricultural, and medical applications [60]. Unlike many organic pollutants, heavy metals are nonbiodegradable, persist in the environment, and bioaccumulate in human tissues, resulting in chronic internal exposure even after external sources are removed [61,62]. The kidney is a primary target organ for metal-induced nephrotoxicity owing to its central role in metal filtration, reabsorption, and sequestration, particularly within proximal tubular epithelial cells [63,64].

### 3.1. Cadmium

Cadmium is one of the most potent and well-characterized nephrotoxic metals. Environmental exposure primarily occurs through contaminated food, drinking water, tobacco smoke, and industrial emissions. Once absorbed, cadmium accumulates in the renal cortex, with a biological half-life exceeding two decades, leading to progressive tubular injury [65,66].

Epidemiological studies have consistently demonstrated associations between low-level cadmium exposure and biomarkers of proximal tubular dysfunction, including increased urinary β_2_-microglobulin, kidney injury molecule-1 (KIM-1), and N-acetyl-β-D-glucosaminidase (NAG) [67,68,69,70]. Additionally, cadmium exposure correlates with reduced eGFR and higher CKD prevalence. Notably, adverse renal effects have been observed at exposure levels below current regulatory limits, suggesting that existing regulatory standards may not adequately protect kidney health [71].

Mechanistically, cadmium induces oxidative stress, mitochondrial dysfunction, and disruption of metal transporters in tubular epithelial cells, triggering inflammatory and fibrotic signaling pathways that promote CKD progression [72,73].

### 3.2. Lead

Lead exposure remains a significant public health threat despite reductions in occupational and environmental sources in many regions [74]. Chronic low-level lead exposure has been linked to tubulointerstitial nephropathy, hypertension-mediated renal impairment, accelerated eGFR decline, and increased kidney-related mortality [75,76,77,78].

Lead accumulates in bone and soft tissues and is mobilized during periods of physiological stress, resulting in prolonged renal exposure, even after the cessation of external exposure. Experimental and clinical studies have suggested that lead induces renal injury through oxidative stress, impaired nitric oxide signaling, endothelial dysfunction, and direct tubular toxicity [79,80,81].

### 3.3. Mercury, Uranium, and Other Nephrotoxic Metals

Mercury exposure, particularly inorganic mercury and methylmercury, has been consistently associated with increased kidney disease mortality and a spectrum of immune-mediated renal injuries, including glomerulonephritis and tubular dysfunction [82,83,84,85]. Chronic exposure exacerbates oxidative stress, mitochondrial dysfunction, and dysregulation of inflammatory pathways. These mechanisms collectively contribute to progressive renal damage [22,86,87].

Human exposure to uranium, which occurs predominantly in contaminated groundwater in mining areas or natural deposits, exerts nephrotoxicity primarily via chemical toxicity rather than radiological effects [88]. Experimental and human studies have indicated that uranium accumulates in proximal tubular cells, leading to direct cytotoxicity, disruption of solute transport, and subsequent decline in renal function [89,90].

Other nephrotoxic metals, including nickel and arsenic, have also been implicated in renal injury [91]. Nickel exposure has been associated with oxidative stress–mediated tubular injury and mitochondrial damage [92,93,94], while arsenic, a common groundwater contaminant, has been shown to contributes to endothelial dysfunction, interstitial fibrosis, and chronic kidney disease progression through both oxidative and inflammatory mechanisms [95,96].

Although population-level epidemiological evidence for these metals remains less extensive than for cadmium and lead, cumulative data support their consideration in environmental and occupational nephrotoxicity risk assessments. Integrating these metals into multi-pollutant risk models is critical in regard to accurately evaluating their contribution to CKD burden and informing preventive public health strategies.

### 3.4. Metal Mixtures and Cumulative Nephrotoxicity

Humans are rarely exposed to a single metal in isolation; real-world exposure typically involves complex mixtures of metals that exhibit convergent pathways of toxicokinetics, molecular targets, and renal accumulation profiles. Emerging epidemiological and mechanistic studies indicate that combined exposure to multiple nephrotoxic metals can produce additive or even synergistic effects on kidney injury. These effects may occur even at subthreshold levels of individual metal concentrations [97,98]. These interactions potentiate oxidative stress, inflammation, and tubular cytotoxicity, thereby accelerating the onset and progression of AKI and CKD.

Recent mixture analyses have demonstrated that the cumulative metal burden correlates more strongly with CKD prevalence, impaired glomerular filtration, and abnormalities in renal biomarkers than single metal exposure alone [99,100]. These findings challenge conventional regulatory paradigms that focus on individual chemicals and underscore the necessity of adopting mixture-based risk assessment approaches in regard to environmental nephrology. Incorporating the complexities of coexposure into public health frameworks is critical for accurately quantifying population-level kidney disease risks and developing effective preventive strategies.

### 3.5. Susceptibility, Life-Course Exposure, and Environmental Justice

Susceptibility to metal-induced kidney injury exhibits significant interindividual variability across populations, reflecting differences in age, life-stage-specific physiological changes, comorbidities, and genetic backgrounds. Children, pregnant individuals, older adults, and those with diabetes or pre-existing CKD are particularly vulnerable to altered metal absorption, distribution, and excretion, as well as diminished renal functional reserve [55,101]. Genetic polymorphisms influencing metal transport and detoxification pathways act as critical modifiers of individual susceptibility, accounting for the variability in renal outcomes even among individuals with similar exposure levels [102,103].

Life-course exposure is a critical determinant of kidney health. Prenatal and early life exposure to nephrotoxic metals can disrupt nephrogenesis, alter renal developmental programming, and increase the risk of CKD later in life. Cumulative exposure during childhood, adulthood, and aging can exacerbate renal decline, particularly when combined with hypertension or metabolic disorders.

From an environmental justice perspective, the burden of metal exposure is skewed towards populations residing near industrial sites, mining regions, and areas with contaminated drinking water, thereby amplifying health disparities. Occupational exposure remains a significant concern in both low- and middle-income countries, where regulatory oversight and protective measures are often insufficient [58,59]. Mitigating these disparities requires targeted environmental monitoring, equitable regulatory enforcement, and policies that prioritize the protection of high-risk and marginalized communities.

### 3.6. Public Health and Policy Implications

Heavy metal–associated kidney disease illustrates the long latency, cumulative toxicity, and often the irreversible nature of environmentally induced renal injury. Because metals can bioaccumulate, and renal damage may progress long after exposure has ceased, primary prevention through stringent environmental regulations, continuous monitoring of food and water safety, and robust occupational protection are paramount [61].

Policy frameworks that integrate kidney-specific endpoints, including the biomarkers of tubular and glomerular injury, and adopt mixture-based risk assessment strategies can accurately capture population-level risks and substantially reduce the future burden of CKD. Environmental monitoring programs should prioritize high-risk communities and life-course exposures and also incorporate geospatial surveillance to identify hotspots of metal contamination.

From a clinical perspective, recognizing environmental metal exposure as a preventable driver provides opportunities for early detection, risk stratification, and targeted intervention in vulnerable populations. Integrating environmental exposure histories into routine nephrology practice, along with conventional risk factor assessments, could facilitate personalized prevention strategies and mitigate the long-term impact of nephrotoxic metals on kidney health.

## 4. Organic Chemical Pollutants

Organic chemical pollutants represent a major and increasingly recognized class of environmental nephrotoxins, extending the scope of environmental kidney injury beyond metals and air pollution to include endocrine-disrupting chemicals, persistent organic pollutants, and industrial compounds with widespread human exposure. These compounds, including persistent organic pollutants (POPs), endocrine-disrupting chemicals, industrial chemicals, and food-related contaminants, are characterized by widespread human exposure, complex toxicokinetics, and the capacity to disrupt multiple biological pathways relevant to renal health [104,105]. Unlike many heavy metals, organic chemicals often exert toxicity through receptor-mediated signaling, metabolic interference, and endocrine disruption, leading to a subtle but sustained renal injury.

Epidemiological and experimental evidence has linked these exposures to reduced kidney function, albuminuria, and CKD progression. Mechanistically, organic pollutants induce oxidative stress, impair mitochondrial function, disrupt hormonal and metabolic signaling, and activate pro-inflammatory and profibrotic pathways in both renal tubular and glomerular cells [106,107,108,109,110]. Given their ubiquity, persistence, and potential for long-term bioaccumulation, organic chemical pollutants represent a substantial yet modifiable risk factor for kidney disease across the full spectrum of renal compartments.

### 4.1. Persistent Organic Pollutants (POPs)

Persistent organic pollutants, including polychlorinated biphenyls (PCBs), dioxins, and organochlorine pesticides, are highly lipophilic, resistant to environmental degradation, and bioaccumulate over time, resulting in chronic internal exposure [111]. Epidemiological studies have consistently linked POP exposure to decreased eGFR and increased prevalence of CKD [108,112]. Mechanistically, POPs are associated with oxidative stress and disruptions in mitochondrial function and redox homeostasis, which underlie cellular dysfunction observed in experimental models and population studies [113].

### 4.2. Endocrine-Disrupting Chemicals (EDCs)

Endocrine-disrupting chemicals, such as phthalates and bisphenol A (BPA), are ubiquitous in consumer products, food packaging, and medical devices. These chemicals result in pervasive and chronic human exposure. Epidemiological studies have linked urinary concentrations of phthalate metabolites and BPA to reduced eGFR, albuminuria, and markers of tubular injury in both adults and children [104,114,115].

Experimental studies have demonstrated that EDCs induce oxidative stress, impair mitochondrial function, and activate fibrogenic cascades in renal tubular epithelial cells [116]. Importantly, EDCs may induce kidney toxicity at low doses and during critical developmental windows, supporting the concept of developmental programming in kidney diseases [117,118].

### 4.3. Per- and Polyfluoroalkyl Substances (PFAS)

PFAS are a class of highly persistent organic pollutants widely used in industrial and consumer applications [119,120,121]. PFASs accumulate in human tissues, including the kidneys, because of their resistance to degradation and protracted biological half-lives. Epidemiological studies have associated PFAS exposure with reduced kidney function and increased risk of CKD. The effect sizes vary across compounds and populations [105,122,123,124].

Mechanistic studies have suggested that PFAS disrupt lipid metabolism, induce mitochondrial dysfunction, and activate inflammatory pathways in renal cells [125,126,127]. Renal tubular transporters have been shown to play key roles in the renal handling of PFAS, potentially leading to selective tubular accumulation and subsequent injury [128,129,130].

### 4.4. Pesticides and Industrial Chemicals

Occupational and environmental exposure to pesticides has been associated with increased risks of AKI, CKD, and CKD of unknown origin (CKDu), particularly among agricultural workers [131,132,133]. Certain pesticides exert direct tubular toxicity, whereas others induce renal injury via oxidative stress and immune-mediated mechanisms [134,135,136,137]. Industrial solvents and chemicals, including hydrocarbons and plasticizers, have also been implicated in kidney injury, although epidemiological evidence remains heterogeneous [138,139]. Importantly, these exposures often co-occur with heat stress and dehydration. This combination may potentiate the nephrotoxicity of these agents in vulnerable populations [140].

### 4.5. Food-Related Organic Contaminants

Food contamination is an important but under-recognized source of organic nephrotoxins. Ochratoxin A, a mycotoxin produced by *Aspergillus* and *Penicillium* species, induces tubular toxicity and fibrotic signaling at nanomolar concentrations, and it has been linked to chronic kidney injury in both experimental and observational studies [141,142,143].

Melamine contamination of food products is associated with kidney stone formation, tubular obstruction, and acute and chronic kidney injury, particularly in infants and young children [144,145,146,147]. These events underscore the vulnerability of the developing kidneys to exogenous chemical exposure.

### 4.6. Mixture Effects and the Exposome

Humans are exposed at the same time to multiple organic chemicals with overlapping and interacting biological effects. Emerging evidence in the literature has suggested that combined exposure to EDCs, PFAS, pesticides, and other organic pollutants may produce additive or synergistic nephrotoxicity, even when the individual chemical concentrations are below regulatory thresholds [118,148,149].

The exposome framework emphasizes the cumulative impact of lifelong exposure to chemical mixtures by integrating environmental, occupational, dietary, and lifestyle factors. Applying exposomic approaches to kidney disease research may improve causal inference and identify modifiable environmental determinants of renal injury [150,151].

### 4.7. Susceptibility and Life-Course Considerations

Susceptibility to organic chemical–induced kidney injury varies with age, sex, genetic background, and physiological state. Prenatal and early life exposures may permanently alter nephron endowment and renal resilience, increasing the lifetime risk of CKD [19,21,109]. Individuals with diabetes, hypertension, or preexisting CKD may experience amplified renal toxicity due to impaired detoxification and a reduced renal reserve [55].

### 4.8. Public Health and Policy Implications

The widespread use and persistence of organic chemical pollutants pose significant challenges in kidney disease prevention. Regulatory frameworks often evaluate chemicals individually and focus on overt rather than subtle long-term renal effects. The incorporation of kidney-specific endpoints, mixture toxicity, and life-course vulnerability into chemical safety assessments can substantially strengthen public health protection [152].

From a clinical perspective, recognizing organic chemical exposure as a modifiable risk factor may inform early detection, risk stratification, and preventive strategies, particularly in regard to highly exposed and vulnerable populations.

## 5. Emerging Contaminants: Microplastics and Nanomaterials

Emerging contaminants, including microplastics and engineered nanomaterials, represent a rapidly expanding and largely unregulated class of environmental exposure with potential implications for kidney health. Unlike traditional pollutants, these materials are defined not only by their chemical composition, but also by their physical properties, such as size, surface charge, and shape, which influence their biological behavior and toxicity [153]. The kidney, a major filtration and excretory organ, is increasingly recognized as a potential target for these novel contaminants.

### 5.1. Microplastics

Microplastics (plastic particles smaller than 5 μm) are ubiquitous in the air, water, food, and consumer products, resulting in continuous human exposure through inhalation and ingestion. Recent studies have detected microplastic particles in the human blood, lung tissue, and other organs, raising concerns about their systemic distribution and organ-specific toxicity [153,154].

Experimental studies have indicated that microplastics can translocate across biological barriers, accumulate in the kidneys, and induce renal injury. In animal and in vitro models, microplastic exposure has been shown to promote oxidative stress, inflammatory responses, and mitochondrial dysfunction. These changes ultimately lead to fibrotic remodeling of renal tissue [153,155]. Additionally, microplastics may act as vectors for other toxic substances, including heavy metals and organic pollutants, thereby amplifying the nephrotoxic potential through combined exposure [156,157].

Although direct epidemiological evidence linking microplastics to kidney disease in humans is limited, the convergence of experimental toxicity data and widespread exposure underscores the plausibility of microplastic-associated renal risk, particularly in the context of chronic low-dose exposure throughout life.

### 5.2. Nanomaterials

Engineered nanomaterials (ENMs), including metal-based nanoparticles, carbon nanotubes, and polymeric nanomaterials, are increasingly used in industrial, medical, and consumer applications. Their nanoscale size enables renal filtration and cellular uptake, distinguishing them from larger particulate pollutants [158].

Experimental evidence suggests that certain nanomaterials can accumulate in the renal tissue and induce dose-dependent nephrotoxicity. The proposed mechanisms include the generation of reactive oxygen species (ROS) and disruption of mitochondrial function. Lysosomal damage and activation of inflammatory and apoptotic pathways in renal tubular cells also contribute to nephrotoxicity [159,160]. Surface modification and physicochemical properties strongly influence renal handling and toxicity, complicating the risk assessment.

Although nanomaterials are also being explored for beneficial biomedical applications, including drug delivery and imaging, concerns remain regarding unintended renal toxicity following environmental or occupational exposure, particularly repeated or poorly characterized exposure.

### 5.3. Interaction with Other Environmental Pollutants

Emerging contaminants do not exist in isolation. Microplastics and nanomaterials may interact with coexisting environmental pollutants, including heavy metals, PFAS, and pesticides, thereby altering their bioavailability and toxicity. In particular, microplastics can adsorb and concentrate hydrophobic organic compounds and metals on their surface, facilitating their transport and renal delivery [161,162]. Such interactions align with the exposome concept, emphasizing that the kidney is exposed to dynamic mixtures of chemical and physical stressors rather than to single agents [151].

### 5.4. Knowledge Gaps and Research Challenges

Despite growing concerns, substantial knowledge gaps remain regarding exposure assessment, dose–response relationships, and long-term renal effects. Standardized methods for quantifying exposure to biological samples are lacking, and human epidemiological data remain sparse [162]. Additionally, most experimental studies have employed exposure levels that may not accurately reflect real-world environmental conditions. Bridging this translational gap requires interdisciplinary approaches that integrate toxicology, nephrology, environmental sciences, and exposure biology.

### 5.5. Public Health and Policy Implications

The rapid proliferation of microplastics and nanomaterials presents a significant regulatory challenge. Current policies are ill-equipped to address particle-based contaminants, which are defined by their physical properties rather than their molecular composition. Incorporating kidney-specific endpoints and life-course vulnerability into regulatory assessments could substantially mitigate future CKD burden. From a precautionary standpoint, reducing plastic production and strengthening occupational protection represent pragmatic strategies for limiting plastic exposure while scientific evidence continues to evolve.

## 6. Biological Mechanisms Linking Environmental Pollution to Kidney Injury

Environmental pollutants include chemically and physically diverse agents. However, their renal effects converge on a limited number of shared biological pathways that drive the initiation and progression of kidney injury. These mechanisms operate at the molecular, cellular, and tissue levels, linking chronic low-level exposure to subclinical renal dysfunction, structural damage, and ultimately CKD [43,44]. Understanding these convergent pathways is essential for integrating heterogeneous environmental exposures into a unified framework for environmental nephrotoxicity (Table 1).

### 6.1. Oxidative Stress and Redox Imbalance

Oxidative stress is a central unifying mechanism. Air pollutants, heavy metals, organic chemicals, and particle-based contaminants promote excessive generation of ROS either directly or through the activation of inflammatory and metabolic pathways [109,163]. The kidney, particularly the proximal tubular epithelial cells, is highly susceptible to oxidative damage because of its high metabolic activity and mitochondrial density. Excessive ROS disrupts cellular redox homeostasis, leading to lipid peroxidation, protein modification, and DNA damage [12,26].

### 6.2. Inflammation and Immune Dysregulation

Environmental pollutants induce local renal inflammation and systemic immune activation. Inhaled particulate matter and gaseous pollutants trigger pulmonary inflammation, resulting in the systemic dissemination (or spillover) of pro-inflammatory cytokines, chemokines, and mediators that reach the kidneys via circulation [23]. Similarly, metals and organic chemicals activate innate immune pathways within renal cells, including NF-κB and inflammasome signaling [164,165]. Persistent low-grade inflammation promotes leukocyte infiltration, tubular–interstitial crosstalk, and sustained activation of fibroblasts, creating a profibrotic microenvironment that accelerates CKD progression [46].

### 6.3. Endothelial Dysfunction and Renal Microvascular Injury

The renal microvasculature is a critical target of environmental toxicity. Environmental pollutants reduce endothelial nitric oxide bioavailability, increase vascular oxidative stress, and disrupt endothelial barrier integrity, leading to impaired renal perfusion and altered autoregulation [166]. Endothelial dysfunction promotes inflammatory activation and microvascular instability in the kidney [23,43]. Persistent endothelial injury results in microvascular rarefaction and capillary loss, resulting in chronic hypoxia in the renal cortex and medulla [167]. This hypoxic environment amplifies tubular injury and activates profibrotic pathways, thereby accelerating renal fibrogenesis and functional decline. Microvascular injury may occur early and potentiate subsequent glomerular and tubular damage. This mechanism is particularly relevant to air pollution and Pb exposure, which are strongly associated with systemic endothelial dysfunction and intrarenal microvascular damage, thereby linking vascular toxicity to structural kidney injury [168,169].

### 6.4. Mitochondrial Dysfunction and Metabolic Disruption

Mitochondrial injury has emerged as a key mechanistic link between environmental pollution and kidney disease [92]. Pollutants disrupt mitochondrial biogenesis, impair oxidative phosphorylation, and induce mitochondrial DNA damage in renal tubular cells [73]. These effects compromise ATP production, increase the generation of ROS, and trigger cell death pathways.

Metabolic dysregulation, including altered lipid handling and glucose metabolism, further sensitizes renal cells to injury, particularly in patients with diabetes or metabolic syndrome [125,170].

### 6.5. Glomerular-Specific Biological Mechanisms of Environmental Kidney Injury

Although much of the mechanistic literature on environmental nephrotoxicity has traditionally emphasized tubular injury, increasing evidence indicates that environmental pollutants also disrupt key biological processes within the glomerular compartment [171,172,173]. Air pollutants, heavy metals, and organic toxicants induce oxidative stress, inflammatory signaling, and endothelial dysfunction in glomerular endothelial cells, podocytes, and mesangial cells, cell types that collectively maintain the integrity of the glomerular filtration barrier [30,174,175].

Experimental and translational studies demonstrate that pollutant-induced ROS and pro-inflammatory cytokines impair podocyte mitochondrial function. They also destabilize slit diaphragm proteins and promote cytoskeletal remodeling, ultimately leading to podocyte loss and increased glomerular permeability [176,177].

Environmental exposure to ambient air pollution has been associated with systemic endothelial dysfunction and markers of impaired vascular homeostasis, including altered nitric oxide bioavailability and increased oxidative stress. These effects may plausibly compromise glomerular microvascular integrity. Long-term exposure to PM_2.5_, NO_2_, and other pollutants has been consistently linked epidemiologically to albuminuria and declines in estimated glomerular filtration rate, suggesting that perturbations in endothelial function contribute to progressive filtration barrier dysfunction in susceptible individuals [33,36,169,178,179]

Heavy metals such as lead and cadmium have been associated with adverse effects on renal function, including decreased estimated glomerular filtration rate, likely mediated through direct nephrotoxic effects on glomerular and tubular cells and oxidative stress pathways. Chronic environmental exposure to these metals correlates with reduced eGFR in general population cohorts and environmentally exposed communities, supporting their contributory role in kidney disease risk [180,181,182].

### 6.6. Tubular Transporter–Mediated Toxicity and Bioaccumulation

Many environmental nephrotoxins exploit the renal tubular transport systems for cellular entry. Heavy metals (cadmium, uranium) and organic anions (PFAS) are taken up by proximal tubular transporters, leading to selective intracellular accumulation. This explains the disproportionate vulnerability of the kidneys to low-level environmental exposures [105,183].

### 6.7. Epigenetic Modification and Developmental Programming

Emerging evidence has suggested that environmental pollutants induce epigenetic alterations such as DNA methylation changes, histone modifications, and microRNA dysregulation, which persist beyond the exposure period and modify long-term kidney disease risk [184,185]. Prenatal and early life exposure may impair nephron endowment and renal resilience, predisposing individuals to CKD later in life [21,186]. These mechanisms provide a biological basis for the life-course vulnerability and transgenerational effects of environmental pollution on kidney health.

### 6.8. Fibrosis as a Final Common Pathway

Regardless of the initiating pollutant, progressive injury ultimately converges into renal fibrosis [187]. Chronic oxidative stress, inflammation, and tubular cell injury activate profibrotic pathways, including transforming growth factor-β signaling and extracellular matrix deposition. Fibrosis represents an irreversible structural correlate of cumulative environmental injury and serves as a critical link between environmental exposure and long-term loss of kidney function [188].

### 6.9. Integrative Conceptual Framework

Collectively, these mechanisms illustrate how diverse environmental pollutants converge on shared renal-injury pathways and produce additive and synergistic effects over time. This mechanistic convergence supports the application of the exposome framework and highlights the opportunities to identify common biomarkers and therapeutic targets across different pollutant classes [151].

## 7. Future Directions and Clinical Implications

Despite accumulating evidence linking environmental pollution to kidney disease, major gaps still remain in regard to exposure assessment, mechanistic understanding, and translation into action. Addressing these gaps requires multidisciplinary coordination among nephrology, environmental health, and public policies.

### 7.1. Advancing Exposure Science and Causal Inference

Future research should prioritize high-resolution exposure assessments that capture the spatiotemporal variability and cumulative lifetime exposure. Integrating satellite-based models and biomonitoring will improve characterization at both individual and population levels [151,189]. Longitudinal cohorts and natural experiments, such as policy-driven pollution reduction, are essential for strengthening causal inferences and delineating critical windows of susceptibility [33].

### 7.2. Integrating Multi-Omics and Mechanistic Approaches

Multi-omics offers powerful tools for elucidating the molecular signatures of injury. Linking omics data with environmental profiles may help to identify early biomarkers and shared pathways across pollutant classes [190,191]. Gene–environment (G × E) interaction studies, involving variants like *APOL1*, highlight the potential for precision environmental nephrology [57].

### 7.3. Mixture Toxicology and the Exposome Framework

However, traditional single-pollutant approaches are insufficient. Future studies should explicitly model pollutant mixtures by using exposure-based frameworks. This is critical because the additive and synergistic effects across diverse contaminants converge on shared injury pathways [151,192,193].

### 7.4. Implications for Clinical Nephrology Practice

Environmental pollution should be recognized as a modifiable and routinely assessable risk factor in nephrology practice. For patients with unexplained CKD, rapid disease progression, or discordance between traditional risk factors and disease severity, a structured environmental exposure history should be incorporated. This includes occupational exposure, residential proximity to pollution sources, and contaminated water or food intake. Where relevant, collaboration with occupational medicine and environmental health specialists may facilitate exposure mitigation and secondary prevention. Such approaches align environmental risk assessment with precision nephrology and may improve early detection and individualized management strategies.

### 7.5. Public Health, Policy, and Systems-Level Interventions

Evidence that kidney injury occurs at pollutant concentrations below current regulatory thresholds raises concern that existing environmental standards may inadequately protect renal health [194,195]. Incorporating kidney-specific endpoints, such as albuminuria, subclinical declines in estimated glomerular filtration rate, and long term risk of CKD, into environmental health impact assessments represents a measurable and actionable policy goal [10,196].

Importantly, the burden of environmentally mediated kidney disease disproportionately affects socioeconomically disadvantaged communities, racial and ethnic minorities, and populations in low- and middle-income countries, reflecting structural environmental injustice [197,198,199,200]. Policies aimed at reducing pollution exposure in high-risk communities, improving water and food safety, and strengthening occupational protections are therefore central to equitable kidney disease prevention and long term population health resilience [63,201,202].

## 8. Conclusions

Environmental pollution is a major, pervasive, and long-underestimated determinant of kidney disease worldwide. The evidence presented in this review has indicated that chemically and physically diverse environmental agents, including ambient air pollutants, heavy metals, organic contaminants, and emerging industrial chemicals, exert convergent effects on renal structure and function. Despite the marked heterogeneity in exposure sources and chemical properties, these agents engage in a shared set of pathogenic pathways. Oxidative stress, inflammatory signaling, endothelial dysfunction, and mitochondrial injury are common mechanistic nodes that promote sustained renal injury and maladaptive repair, ultimately driving fibrosis and CKD progression. This mechanistic convergence provides a unifying explanation as to why distinct environmental exposures result in similar renal phenotypes across different populations.

While global life expectancy has increased, environmental pollution contributes to a shift from healthy aging toward prolonged morbidity, accelerating the onset and progression of chronic kidney disease and amplifying multimorbidity and frailty. Thus, the consequence of environmental degradation is not reduced longevity in itself, but an expanding burden of chronic disease, disability, and health inequity.

A central implication of these findings is that environmental pollution is a modifiable and policy-responsive risk factor for kidney disease. Viewing kidney disease through an environmental lens expands prevention beyond individual-level clinical management and situates nephrology within the broader domains of public and planetary health. Interventions that reduce pollutant emissions, improve environmental quality, and address exposure inequities have the potential to yield substantial downstream benefits for kidney health with population-level reductions in CKD incidence and progression.

As the global burden of kidney disease continues to increase, the incorporation of environmental determinants into research frameworks, clinical decision-making, and health policies is no longer discretionary. Progress will require coordinated efforts in order to integrate mechanistic biology with high-resolution exposure assessments and evidence-based policy actions. This interdisciplinary approach offers a realistic and sustainable pathway towards mitigating the risk of kidney disease in an increasingly polluted environment.

## Figures and Tables

**Table 1:** Summary of Environmental Pollutants and Their Impact on Kidney.

Category	Key Pollutants	Primary Exposure Routes	Mechanisms of Renal Injury	Clinical/Renal Outcomes
Air Pollution	PM_2.5_, NO_2_, SO_2_	Inhalation	Systemic inflammation, oxidative stress, endothelial dysfunction	Reduced eGFR, CKD progression, Albuminuria
Heavy Metals	Lead, Cadmium, Arsenic, Uranium	Ingestion (water/food), Occupational	Proximal tubular accumulation, mitochondrial damage, oxidative stress	Tubular dysfunction, Fanconi-like syndrome, CKD
Organic Pollutants	PFAS, Phthalates, BPA, Pesticides	Consumer products, Food/Water, Occupational	Receptor-mediated signaling, endocrine disruption, metabolic interference	Reduced eGFR, CKD/CKDu, tubular injury markers
Food Contaminants	Ochratoxin A, Melamine	Ingestion (contaminated food)	Fibrotic signaling, crystal formation, tubular obstruction	Kidney stones, AKI, Chronic tubulointerstitial fibrosis
Emerging Contaminants	Microplastics, Nanomaterials	Ingestion, Inhalation, Medical	Translocation across barriers, vector effects (carrying other toxins)	Potential tubular accumulation, oxidative stress, fibrosis

## Data Availability

No new data were created or analyzed in this study.
